# A *Very* Late Viral Protein Triggers the Lytic Release of SV40

**DOI:** 10.1371/journal.ppat.0030098

**Published:** 2007-07-20

**Authors:** Robert Daniels, Dorota Sadowicz, Daniel N Hebert

**Affiliations:** 1 Department of Biochemistry and Molecular Biology, University of Massachusetts, Amherst, Massachusetts, United States of America; 2 Program in Molecular and Cellular Biology, University of Massachusetts, Amherst, Massachusetts, United States of America; Northwestern University, United States of America

## Abstract

How nonenveloped viruses such as simian virus 40 (SV40) trigger the lytic release of their progeny is poorly understood. Here, we demonstrate that SV40 expresses a novel later protein termed VP4 that triggers the timely lytic release of its progeny. Like VP3, VP4 synthesis initiates from a downstream AUG start codon within the VP2 transcript and localizes to the nucleus. However, VP4 expression occurs ∼24 h later at a time that coincides with cell lysis, and it is not incorporated into mature virions. Mutation of the VP4 initiation codon from the SV40 genome delayed lysis by 2 d and reduced infectious particle release. Furthermore, the co-expression of VP4 and VP3, but not their individual expression, recapitulated cell lysis in bacteria. Thus, SV40 regulates its life cycle by the later temporal expression of VP4, which results in cell lysis and enables the 50-nm virus to exit the cell. This study also demonstrates how viruses can generate multiple proteins with diverse functions and localizations from a single reading frame.

## Author Summary

The release of viral particles from an infected host cell is essential for a virus to spread within the host organism. Cytolytic viruses such as the common cold, poliovirus, and simian virus 40 (SV40) release their progeny by inducing lysis or death of the host cell. For efficient viral spreading, it is critical that optimal numbers of the virus are assembled before cell lysis and release occurs. Therefore, the timing of cell lysis is an integral and well-controlled step in the viral life cycle. For many years, lysis has been thought to be a nonspecific consequence of viral protein overexpression and the massive production of viral progeny. As SV40 was the first mammalian virus sequenced almost 30 years ago, it is an excellent model virus for investigating the poorly understood mechanism of viral release. In this study, we have identified a novel SV40 protein named VP4 that is required for the timely lytic death of the host cell, and hence regulates the spread of SV40. The late expression of VP4 offers a sufficient period for virion assembly to occur before it initiates the lytic release of the newly assembled viral progeny.

## Introduction

Eukaryotic cells use a range of mechanisms and pathways to shuttle molecules across their complex endomembrane network [1]. Viruses exploit these pathways to gain entry into the cell and navigate through the various membrane barriers [2,3]. However, no known cellular pathways exist for exporting nonenveloped DNA viral progeny from their intracellular site of assembly to the extracellular milieu. Therefore, nonenveloped viruses, including simian virus 40 (SV40), initiate their release from the host cell in a lytic manner. How these cytolytic viruses kill the host cell and rupture its membrane system to facilitate release remains largely unknown [4].

For many years, viral-induced cell lysis was thought to be a nonspecific consequence of viral protein overexpression or the production of large amounts of viral progeny causing changes in cell membrane permeability. However, the necessity for the lytic event to occur in a timely fashion after the completion of viral assembly suggests that cytolytic viruses have evolved a method to ensure that the induction of cell death takes place at the proper time. The enterovirus 2B protein, for example, is proposed to aid in the lytic release process due to its ability to induce calcium leakage from the endoplasmic reticulum (ER) and alter cell permeability [5]. The adenovirus death protein has also been implicated in viral release because its removal significantly prolongs the adenoviral life cycle [6]. In SV40, the core structural proteins VP2 and VP3 are capable of permeabilizing bacterial membranes and post-translationally integrating into ER membranes in a VP1-regulated manner [7,8]. While these data are supportive of VP2 and VP3 potentially playing a role in the lytic release of SV40, it remains to be determined if other viral or host cellular proteins are involved in this process.

SV40 encodes two early gene products (small and large T antigens), four late gene products (VP1, VP2, VP3, and agno), and microRNAs that help regulate the temporal expression of these proteins [9–11]. In this manuscript, we have identified a new SV40 gene product called VP4 that is expressed ∼24 h after the late structural proteins VP1, VP2, and VP3. VP4 is essential for the timely lytic release of the viral progeny that enables the efficient spreading of SV40 in culture. VP4 oligomerizes with VP3, and when expressed together, these two proteins possess a lethal lytic property that is conserved in bacteria. These data demonstrate that late in the replication process, following viral assembly, SV40 expresses VP4 to initiate the death of the host cell and the efficient release of its progeny.

## Results

### SV40 Expresses an Additional Protein Termed VP4

**Figure 1:**
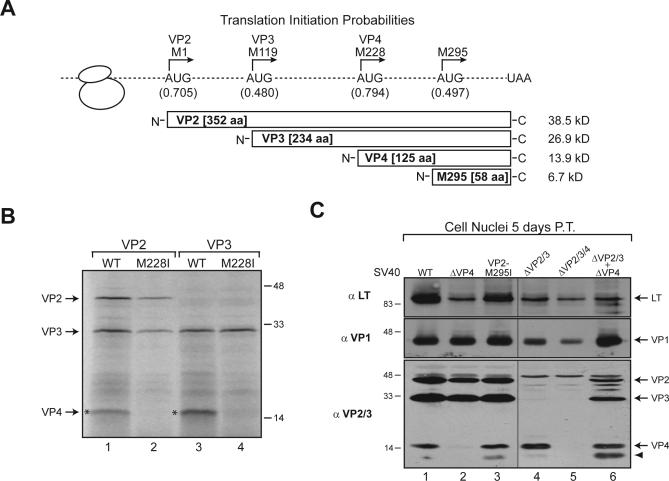
SV40 Expresses an ∼15-kD Protein Termed VP4 (A) Schematic representation of VP2 displaying the translation initiation probabilities of the in-frame AUG initiation codons as predicted by NetStart^1.0^ and the respective molecular weights of the resultant polypeptides [12]. (B) VP2 and VP3 mRNAs express VP4 from Met228 in VP2 upon translation in reticulocyte lysate. (C) VP4 is expressed from Met228 in VP2 during SV40 infection. Immunoblot analysis of the cell nuclei fractions isolated from BS-C-1 cells transfected with the designated viral genomes for 72 h and probed with the indicated antibodies. The arrowhead indicates a band with a faster mobility than VP4 that reacts with VP2 and VP3 antisera.

The in vitro translations of mRNAs encoding VP2 and VP3 from SV40 unexpectedly produced a protein which migrated at ∼15 kD, in addition to VP2 and VP3 (Figure 1B, lanes 1 and 3, asterisks). The coding sequence for VP2 contains four highly conserved in-frame AUG codons (Figures 1A and S1, Met residues). To investigate which of these potential AUG initiation sites was responsible for the synthesis of the smaller protein, an algorithm that predicts AUG initiation sites (NetStart^1.0^) was employed [12]. Surprisingly, the highest probability score for an AUG translation initiation site corresponded to Met228 in VP2, which, if utilized, would yield a protein with a molecular mass of 13.9 kD (Figure 1A, scores in parentheses).

Mutation of the ATG codon for Met228 in VP2 to ATA (Ile) in both VP2 and VP3 prevented the in vitro synthesis of the ∼15-kD protein, which we have termed VP4 (Figure 1B). Note that in later figures, the VP2-Met228Ile mutant is designated as ΔVP4. To investigate whether VP4 was expressed during infection, a series of mutant SV40 genomes were generated in the bacterial replication competent plasmid pSV40 [8,13,14]. In addition, the VP2-M295I genome had the ATG codon corresponding to Met295 (VP2-M295I) in VP2 mutated to ATA (Ile). The ΔVP2/3 genome had both ATG initiation codons for VP2 (Met1) and VP3 (Met119) altered to ATA (Ile), whereas the ΔVP2/3/4 genome had the initiation codons for VP2, VP3, and VP4 altered. The wild-type (WT) and mutant genomes were transfected into permissive BS-C-1 cells. The cells were collected 5 d post-transfection, after VP1, VP2, and VP3 expression, and the post-nuclear and nuclear lysates were analyzed by immunoblotting, because all of these proteins are synthesized in the cytoplasm and imported into the nucleus for viral assembly.

All of the genomes correctly expressed and localized both the early viral protein large T antigen (LT) and the major capsid protein VP1 (Figure 1C). Late viral protein synthesis from the VP2 reading frame was examined by immunoblotting with polyclonal antisera against VP3 (αVP2/3), which also recognizes VP2. Cells transfected with WT SV40 or the VP2-M295I genome expressed three proteins that reacted with VP3 antisera: VP2, VP3, and the ∼15 kD VP4 (Figure 1C, lanes 1 and 3, αVP2/3). In contrast, the ∼15-kD VP4 protein was not observed following transfection with the ΔVP4 and the ΔVP2/3/4 genomes (Figure 1C, lanes 2 and 5, αVP2/3). Importantly, VP4 was synthesized in cells transfected with the ΔVP2/3 genome, which indicates that it was not produced by the proteolytic cleavage of VP2 or VP3. In addition, VP4 predominantly localized to the nucleus upon cellular fractionation, whereas VP2 and VP3 were present in both the post-nuclear and nuclear samples (Figures 1C and S2). Significantly, this indicated that during infection, SV40 expresses the ∼15-kD VP4 protein by initiating its synthesis from Met228 in the VP2 reading frame, resulting in a polypeptide that corresponds to the C-terminal 125 amino acids of VP2 and VP3 (Figure 1A).

### VP4 Is Essential for the Efficient Propagation of SV40 and Is Nuclear Localized

To determine the effects of the various mutations on SV40 propagation, the number of BS-C-1 cells expressing LT over the course of infection was quantified by immunofluorescent microscopy. Contrary to plaque assays, which indirectly measure viral infectivity based on cell death, this method directly monitors viral spreading by determining the number of cells expressing the viral LT [7,8].

**Figure 2:**
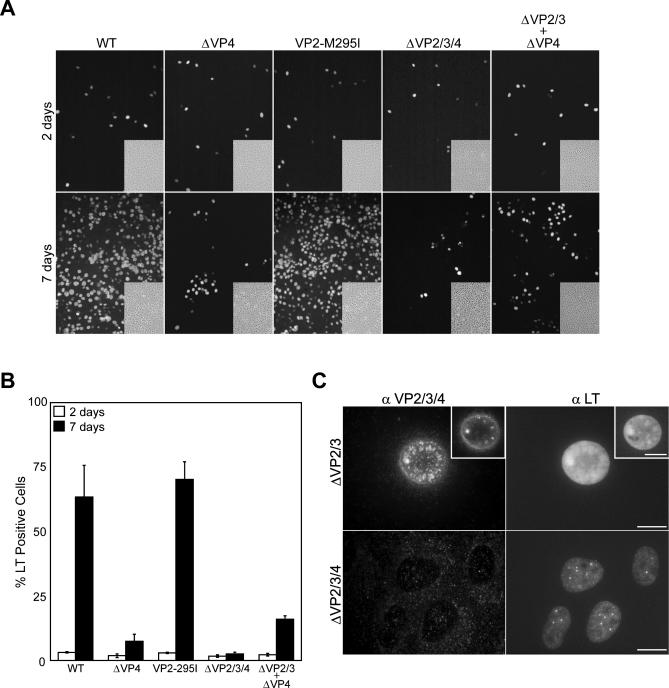
VP4 Is Required for Efficient SV40 Propagation (A) Representative fluorescence images with the corresponding phase-contrast image (inset) of SV40 propagation in BS-C-1 cells following transfection with WT, ΔVP4, VP2-M295I, and ΔVP2/3/4 genomes and co-transfection with the ΔVP2/3 and ΔVP4 genomes. The cells were fixed at the indicated times post-transfection and stained with LT antisera. (B) Quantifications from two replicate experiments showing the percentage out of ∼3,000 BS-C-1 cells that expressed LT at the indicated times post-transfection with the various SV40 genomes. (C) Fluorescent images of BS-C-1 cells transfected with the ΔVP2/3 or ΔVP2/3/4 genomes for 72 h and stained with antisera for VP2/3/4 (αVP2/3/4) or LT (αLT). Compilations of the z-sections are shown as Max projection images, and insets containing a single z-section from the mid-region of the nucleus are included. The bars represent 10 μm, the antibodies used are noted across the top of the composites, and the transfected genomes are listed to the left.

Following transfection, equivalent numbers of primary infections were established from the various genomes as ∼3% of the cells expressed LT after 2 d (Figure 2A and 2B). As expected, the particles generated in cells transfected with the ΔVP2/3/4 genome were incapable of establishing secondary infections. The viral particles produced from the VP2-M295I genome propagated as efficiently as WT, infecting ∼65% of the cells by 7 d, indicating that synthesis of a protein from the AUG codon corresponding to Met295 in VP2 was not required for SV40 infection. In contrast to WT and VP2-M295I, ΔVP4 only infected ∼10% of the cells by 7 d. This demonstrated that VP4 played a role in viral propagation, as limited cell to cell spreading was observed in its absence. In addition, co-transfection of ΔVP2/3 with ΔVP4 partially complemented the ΔVP4 mutation, as ∼20% of the cells were positive for LT by day 7 (Figure 2A and 2B).

Upon cell fractionation, VP4 was predominantly found in the nuclear pellet of cells infected or transfected with the SV40 genome (Figure S2 and unpublished data). To more thoroughly explore the cellular localization of VP4, immunofluorescence microscopy was employed. Because the polyclonal antibody raised to VP3 recognizes VP2, VP3, and VP4, the localization of VP4 was analyzed after transfection with the ΔVP2/3 genome. This enabled any signal from the VP3 antisera to be attributed to VP4 and allowed the localization to be examined in a context similar to that of a viral infection, as LT and VP1 are still expressed. At 3 d post-transfection, VP4 was found to localize to the nucleus, where it was enriched at the inner nuclear membrane periphery and in punctate structures (Figure 2C). In addition, VP4 appeared to support a significant alteration in the nuclear morphology. The nuclei, as defined by LT, showed an obvious circular shape and size increase in cells transfected with the ΔVP2/3 genome, which expresses VP4. This was a drastic change from the cells transfected with the ΔVP2/3/4 genome, where VP4-like immunostaining was not observed and the cell nuclei were a smaller kidney shape (Figure 2C). Thus, VP4 appears to contribute to the expansion of the nucleus that occurs prior to the lytic release of SV40.

### VP4 Is Expressed in the Infected Cell, but Is Not Incorporated into the Viral Particle

**Figure 3:**
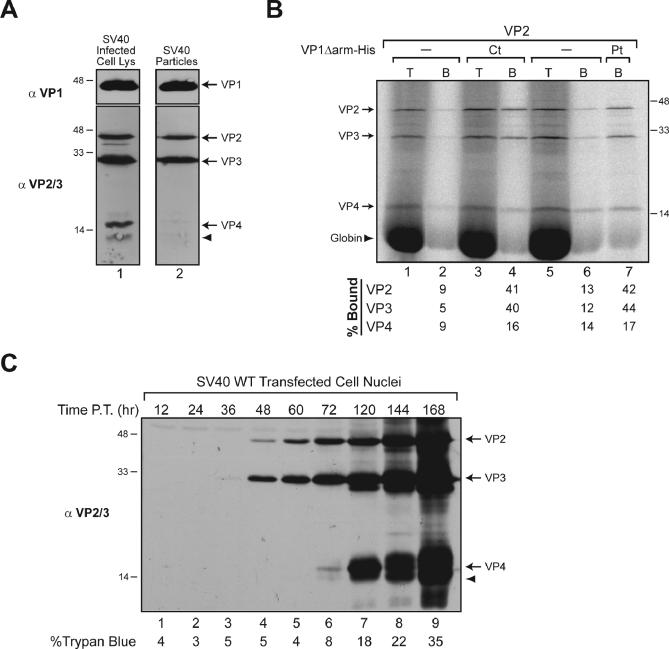
VP4 Is Expressed at the Onset of Lysis and Is Not Incorporated into SV40 Virions (A) VP4 is not incorporated into the mature SV40 virions. Immunoblot analysis of SV40-infected cells and isolated virions with the indicated antisera. (B) VP4 does not associate with VP1 pentamers. VP1 pentamers (VP1Δarm-His) purified from bacteria were either present co-translationally (Ct) or during the synthesis of radiolabeled VP2, VP3, and VP4 in reticulocyte lysate or added post-translationally (Pt). The fractions of VP1-bound (B) VP2, VP3, and VP4 were isolated by Ni-NTA-sepharose beads, washed, and resolved by SDS-PAGE next to an equivalent fraction of the total (T) products. The percentage of the bound (B) fraction from two independent experiments is included. (C) Immunoblot analysis of isolated nuclei from BS-C-1 cells at the indicated time post-transfection with the WT SV40 genome demonstrates that VP4 is expressed at the onset of viral-induced host cell lysis. The percentage of ∼2,000 cells staining positive for trypan blue at each time point is listed.

To elucidate the role of VP4 in the viral life cycle, we initially determined whether VP4 was incorporated into the mature virion, as it contains the proposed VP1 pentamer–binding domains found in VP2 and VP3 [15,16]. Immunoblot analysis with VP2/3 antisera of SV40 virions isolated by filtration and centrifugation indicated that VP4 was not found in SV40 particles, but significant quantities were present in cellular lysates (Figure 3A). Thus, VP4 is a nonstructural viral protein that must perform an integral function in the host cell to promote viral propagation.

To test if the absence of VP4 from the SV40 particles was due to its inability to associate with VP1, binding to VP1 pentamers was examined. VP1 pentamers were produced from bacteria by expressing a construct lacking the VP1 C-terminal arms (VP1Δarm-His), which are necessary for interpentameric association and capsid assembly but are not required for VP2 and VP3 binding [8,17]. In vitro translated radiolabeled VP2, VP3, and VP4 were synthesized in the presence of VP1 pentamers (co-translationally), or incubated with the VP1 pentamers after synthesis was completed (post-translationally).

Following isolation of the VP1 pentamers, a significant fraction of the in vitro translated VP2 and VP3 (∼30% above background) was found to associate with the VP1 pentamers both co- and post-translationally (Figure 3B). In contrast, only slight levels (∼3%–7% above background) of VP4 associated with the VP1 pentamers using either mechanism (Figure 3B). The absence of VP2 or VP4 from the translation reaction had little effect on the association of VP3 with the VP1 pentamers (Figure S3A). Thus, the lack of VP4 binding to VP1 possibly explains why it is not found in the viral particle.

### VP4 Is Synthesized “Later”

To gain further insight into what role VP4 performs in the host cell that contributes to the viral life cycle, its temporal expression was examined in relation to the late proteins. VP2 and VP3 were first observed at 48 h post-transfection (Figure 3C). Strikingly, VP4 initially accumulated at 72 h post-transfection, 12 to 24 h later than the start of VP1, VP2, and VP3 expression (Figure 3C and [7,8]). The temporal separation of VP4 synthesis from viral assembly provided an additional explanation as to why VP4 was excluded from incorporation into SV40 particles. Furthermore, viral-induced cell lysis also initiated at 72 h post-transfection as monitored by the trypan blue staining of cells (Figure 3C, bottom). The requirement of VP4 for efficient SV40 propagation and the finding that it is a nonstructural protein synthesized at later times coinciding with host cell lysis support a function for VP4 in viral-induced lysis of the host cell.

### The Co-Expression of VP4 and VP3 in Bacteria Is Lethal

VP3 expression has been previously shown to induce bacterial lysis by possessing an inherent lytic property, which suggests that VP3 may act as a viroporin [7]. The apparent involvement of VP4 in the lytic release of SV40 led us to investigate whether VP4 expression from the VP3 construct resulted in the previously observed bacterial lysis caused by VP3. To monitor bacterial lysis, the expression of C-terminal His-tagged VP3 (VP3-His), VP3-His with the VP4 initiation codon mutated to ATA (VP3-M228I-His), VP4-His, and the negative control VP1-His was induced with isopropyl-β-D-thiogalactopyranoside (IPTG), and the optical density (OD) at 600 nm of the Escherichia coli suspension was measured.

**Figure 4:**
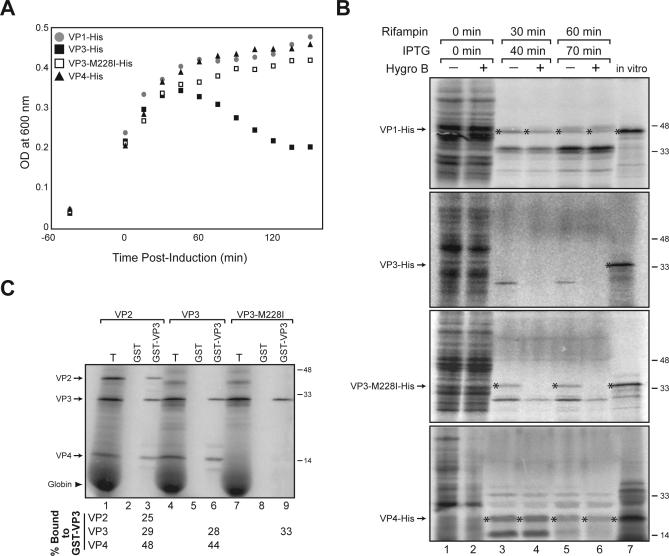
VP4 Expression in Concert with VP3 Induces Bacterial Lysis (A) E. coli viablility was monitored following the induction of VP1-His, VP3-His, VP3Met228I-His, and VP4-His expression. (B) In the absence of VP4, VP3 renders the E. coli permeable to hygromycin B. Analysis of E. coli permeability to hygromycin B during a 10-min pulse at the indicated time post–IPTG induction in the presence of rifampicin. Radiolabeled in vitro synthesized products of each construct were included as references for the expressed protein. Asterisks denote the overexpressed protein. (C) VP4 oligomerizes with VP3. Radiolabeled VP2, VP3, and VP3-M228I were synthesized in reticulocyte lystate prior to GST-VP3 binding and isolation. The percentage of the total (T) bound to GST-VP3 is indicated.

As previously observed, the OD of the E. coli suspension significantly decreased following the induction of VP3-His expression (Figure 4A) [7]. Strikingly, E. coli expressing the VP3-M228I-His construct (without the VP4 initiation codon), or VP4-His alone, remained viable and entered stasis as the OD reached a plateau similar to the OD of VP1-His (Figure 4A). These findings indicate that the co-expression of both VP4 and VP3 is required to induce bacterial lysis.

Next, an assay was utilized to characterize the viroporin activity of the late proteins and determine whether they compromise the bacterial membrane by forming lethal or nonlethal membrane perturbations. E. coli membrane permeability was examined by monitoring the sensitivity of the bacteria to the membrane-impermeable protein synthesis inhibitor hygromycin B following expression of VP3-His, VP3-M228I-His, VP4-His, and the negative control VP1-His (for experimental design, see Figure S4 and [7]).

Prior to induction with IPTG, hygromycin B had little effect on protein synthesis, as the E. coli proteins were readily labeled with ^35^S-Met/Cys (Figure 4B, lanes 1 and 2). At 40 and 70 min post-induction with IPTG, detectable levels of VP3-His were not produced in the absence or presence of hygromycin B. The lack of ^35^S-labeled VP3-His being observed was likely due to the lysis of the E. coli expressing both VP3-His and VP4-His before reaching synthesis levels that were sufficient for visualization (Figure 4A and 4B). In contrast, all of the nonlethal constructs (VP1-His, VP3-M228I-His, and VP4-His) were expressed at detectable quantities in the absence of hygromycin B following IPTG induction (Figure 4B, asterisks). Of the nonlethal constructs (determined from Figure 4A), only VP3M228I-His rendered the bacterial membranes permeable to hygromycin B, as the inhibitor prevented the synthesis of ^35^S-labeled VP3-M228I-His. Together, these findings demonstrate that in the absence of VP4, VP3 is capable of permeabilizing membranes in a nonlethal fashion. However, in the presence of VP4, VP3 has a lethal property that results in membrane lysis.

### VP4 Oligomerization

Small pathogenic proteins that form pores and induce cell lysis generally insert into cellular membranes in an oligomeric state [18]. The observed bacterial lysis following the co-expression of VP4 with VP3, but not the individual expression of either construct, suggested that the lytic property was a result of VP4 oligomerization with VP3. To investigate whether VP4 associates with VP3, VP4 synthesized from VP2 and VP3 mRNAs was monitored for its ability to bind GST-VP3 purified from bacteria. VP4 (∼45% bound) showed a substantially higher affinity for sepharose-bound GST-VP3 than VP2 and VP3 (∼25%–30% bound), the positive control VP1 (∼12% bound), and the negative control luciferase (Figures 4C and S3B). These data imply that the resultant lethality observed in bacteria co-expressing VP3 and VP4 may be due to a change in the physical properties of VP3 as a consequence of its association with VP4.

### Deletion of VP4 Delays the Cytolytic Release of SV40

To determine how the absence of VP4 affected the normal course of infection, SV40 particles were generated from BS-C-1 cells transfected with either the WT SV40 or ΔVP4 genomes. In BS-C-1 cells, both the viral-mediated and transfection-mediated life cycles of SV40 have been well characterized with lengths of 2.5 to 4 d and 3 to 5 d, respectively [7,8]. After transfection, ∼2% of the BS-C-1 cells were infected with each genome as determined by LT expression at 2 d. Trypan blue staining revealed that these low level transfection-initiated ΔVP4 infections required ∼24 d, or the length of six WT life cycles, to lyse the majority of the cell population. This was significantly longer than the similar low level transfection-initiated WT SV40 infection, which required ∼12 d, or three life cycles, to completely lyse a cell population of equivalent size. The viral-containing supernatant was isolated from the ΔVP4-transfected cells at 24 d and the WT-transfected cells at 12 d. The number of LT-expressing BS-C-1 cells was then calculated at 2 d post-infection with equivalent fractions of the ΔVP4 and the WT virus-containing supernatants isolated at 24 and 12 d post-transfection, respectively. Strikingly, ∼95% fewer cells were expressing LT at 2 d post-infection with the ΔVP4 virus-containing supernatant compared to those with the WT virus-containing supernatant.

**Figure 5:**
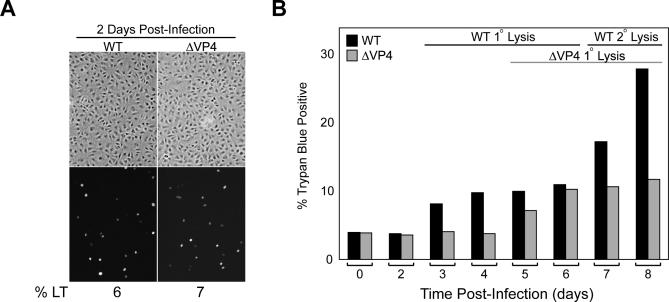
VP4 Directs the Timely Lytic Release of SV40 Progeny (A) Representative fluorescence images and the corresponding phase-contrast images displaying WT and ΔVP4 particle infectivity standardization at 2 d. The cells are stained with LT antisera and the percentage of BS-C-1 cells staining for LT is displayed below the images. (B) Removal of VP4 extends the SV40 life cycle 2 d. Trypan blue staining of ∼2,000 BS-C-1 cells at the indicated times post-infection with equivalent WT and ΔVP4 infectious particles.

The aforementioned deficiencies suggested that the ΔVP4 strain had a prolonged life cycle due to a defect in the lytic release of its viral progeny. To investigate this possibility, BS-C-1 cells infected with equivalent amounts of infectious WT and ΔVP4 particles (Figure 5A, ∼6% LT-positive cells at 2 d) were monitored for viral-induced cell death by trypan blue staining. The primary WT-infected BS-C-1 cells began to die at 3 d post-infection, reaching a plateau at 4 d until the secondary infected cells started to lyse at 7 d (Figure 5B). The primary ΔVP4-infected cells showed a noticeable 2-d lag in lysis, which initiated at ∼5 d and completed by 6–7 d (Figure 5B). Together, these results demonstrate that SV40 expresses a novel later protein (VP4) that initiates the efficient lytic release of its progeny from the host cell.

## Discussion

SV40 has been extensively studied for the past 50 years, yet how it and other noneveloped cytolytic viruses cause cell lysis to facilitate progeny release is relatively unknown. However, new discoveries into the regulation of its temporal viral protein expression have recently been made [11]. Here, we demonstrate that SV40 expresses a very late nonstructural viral protein that we have termed VP4. VP4 is encoded within the VP2 and VP3 transcript from a downstream in-frame AUG start codon, and its expression triggers membrane lysis to aid in viral release for the efficient propagation of SV40.

In support of the hypothesis that VP4 has a role in the lytic process, we observed that VP4 was expressed concurrently with viral-induced lysis ∼24 h after the known late structural proteins, and that its deletion significantly reduced SV40 spreading by prolonging the viral life cycle. Furthermore, SV40-induced cell lysis could be recapitulated in E. coli by co-expressing VP4 and VP3, but not by their individual expression, indicating that SV40-induced lysis likely occurs independently of eukaryotic host factors. Altogether, this suggests that VP4 expression initiates the timely execution of the lytic process through a mechanism that relies on its association with VP3.

There are several reasons that explain why VP4 has gone undetected. First, the small molecular mass of VP4 causes it to migrate off the bottom of most gel separation systems used to visualize the larger structural proteins. Second, VP4 is not incorporated into the viral particle, but is predominantly found in the frequently discarded nuclear fraction. Next, VP4 is expressed very late, immediately before cell lysis. Finally, VP4 is found in lysed cells and is susceptible to proteolysis by trypsin (Figure S2), which is a method commonly used to harvest adherent cells. These properties explain why the identification of VP4 has been elusive and provide valuable insight into its function.

It is unlikely that the observed defect in ΔVP4 propagation was caused by the mutations created in VP2 (Met228 to Ile) and VP3 (Met109 to Ile). The ΔVP2/3 genome, which expresses VP4 and not VP2 and VP3, was able to complement the ΔVP4 genome that contains VP2 and VP3 with the aforementioned Met to Ile mutations. The observed 2-fold rescue in viral propagation following the co-transfection of ΔVP2/3 with ΔVP4 was considered significant due to the fact that ΔVP2/3 has no cell to cell spreading capabilities by itself [7,8]. This further supports the conclusion that VP4 was required for the efficient propagation of SV40.

**Figure 6:**
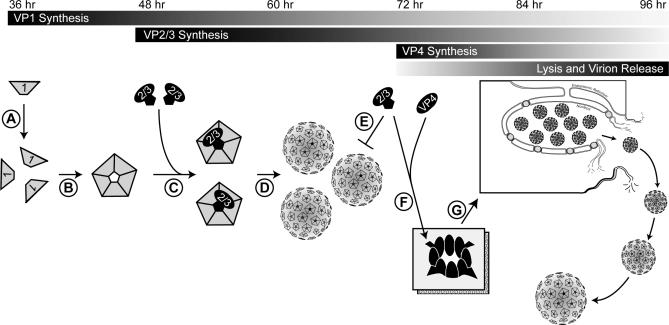
Proposed Model Displaying the Regulation of the SV40 Lytic Cycle VP1 is synthesized first, forming pentamers that sequester newly synthesized VP2 and VP3 (steps A–C). Virion assembly (step D) and the stoichiometry of five VP1 per VP2/3 prevent VP2 and VP3 particle incorporation (step E). VP4 is synthesized, it oligomerizes with VP3, and possibly VP2, and targets to the host cell membrane to form a pore (step F), which then initiates the lytic death of the host cell and the release of the progeny (step G).

Cytolytic viruses such as polyomaviruses (SV40, JCV, BKV) and picornaviruses (poliovirus, rhinovirus, and coxsackievirus) induce host cell lysis to release their progeny and prevent their encapsulation in cellular membranes. However, these viruses must regulate the induction of lysis to ensure that it only occurs after the viral assembly stage. SV40 is known to utilize a series of tightly controlled timing mechanisms to direct its replication. At the onset of infection, the early genes trigger viral genome replication before the synthesis of the structural proteins, which assemble around the genome. During assembly, VP1 acts as a second timer. The expression of VP1 prior to VP2 and VP3 (Figure 6, step A) [8] pre-positions the VP1 pentamers to bind to the newly synthesized VP2 and VP3, preventing their insertion into membranes (steps B and C). These VP1–VP2 and VP1–VP3 complexes are then imported into the nucleus for virion assembly, and the amount of VP2 and VP3 starts to exceed the number of available VP1 pentamers (steps D and E). At this stage, VP4 begins to accumulate, forming hetero-oligomers with VP3, and possibly VP2, which insert into the host cell membranes (Figure S5) to initiate cell lysis and viral release (Figure 6, steps F and G). This model demonstrates how the later expression of VP4 triggers cell lysis.

Viruses are known to evade immune detection and prolong the survival of the infected cell by inhibiting the apoptotic response [19]. However, once the replication process has completed, cytolytic viruses must override both the viral anti-apoptotic and the host apoptotic responses in order to induce the lytic death of the host cell. In SV40-infected cells, LT acts to prevent the apoptotic response by sequestering p53 [20,21]. To counteract the protective activity of LT and induce lysis at the appropriate time, SV40 expresses VP4 very late in assembly. Thus lysis, and hence the length of the SV40 life cycle, is regulated by the temporal expression of VP4, explaining why its removal significantly delayed the lytic death of infected cells.

Upon sequence analysis of the 12 known polyomaviruses from mammalian and avian species, 11 were found to possess a potential VP4 initiation codon at a similar position in their VP2 transcripts. This suggests that VP4 performs a conserved function, but a detailed investigation to determine whether VP4 regulates lysis in other polyomaviruses is necessary. For the lone exception (goose hemorrhagic polyomavirus), the absence of a VP4 initiation codon could explain its lengthy life cycle (7 d until the first observed cytopathic effects in primary goose kidney cells) and the inability to effectively produce the virus in culture [22]. A question of future concern now becomes, how is the later expression profile for VP4 controlled?

The requirement of VP4 to be co-expressed with VP3 in order to recapitulate cell lysis in bacteria indicated that the association of VP4 with VP3 dramatically alters the properties of these two proteins. VP3 is a viral structural protein that is thought to be involved in the penetration process [8]. It is expressed late, binds to VP1 pentamers, forms oligomers, and is able to post-translationally insert into ER membranes. In the case of VP4, the structure appears to be altered in such a way that it hides the VP1-binding domain, explaining why VP4 and VP4-like constructs with small tags that contain the majority of VP4 are not able to bind VP1 [23]. In previous studies where VP1 binding to VP4-like and VP4-truncated constructs containing N-terminal GST-tags was observed, the larger GST-tags may have prevented this change in the conformation of VP4 [15,24]. Comparative analysis of VP2, VP3, and VP4 revealed that these proteins possess distinct hydrophobic properties that likely contribute to their functional and conformational variations (Figure S6 and [8]).

The evolutionary pressure placed on viruses to perform multiple tasks with small genomes has caused viruses to diversify their genomes by encoding a number of proteins within single transcripts. They accomplish this task by organizing the genes as large polyproteins or in a polycistronic manner in which different genes are contained within alternative reading frames or at downstream AUG initiation sites in a single reading frame. Within SV40, VP3 and VP4 are expressed from successive downstream AUG codons in the same reading frame as VP2. This strategy for the creation of N-terminally truncated proteins could also be useful for modifying the role of cellular proteins by removing regulatory or functional binding domains, or by altering their localization by deleting targeting sequences. Through these simple mechanisms, cells and their pathogens can significantly expand and diversify the protein population expressed from their genomes.

## Materials and Methods

### SV40 mutant genomes, cell lines, transfection, and particle isolation.

The bacterial replication competent plasmid pSV40, encoding the entire WT SV40 genome (strain 776), was obtained from H. Kasamatsu (Los Angeles, California, United States). All of the SV40 mutant genomes were created by site-directed mutagenesis (Stratagene, http://www.stratagene.com/) of the ATG codons corresponding to the indicated VP2 Met residues in pSV40 to ATA (Ile) codons. The ΔVP4 and VP2-M295I genomes had Met228 (ΔVP4) or Met295 (VP2-M295I) within the VP2 reading frame altered to ATA (Ile). The ΔVP2/3 genome had the ATG initiation codons for VP2 (Met1) and VP3 (Met119) in VP2 mutated to ATA (Ile). The ΔVP2/3/4 genome contained the additional mutation of the ATG initiation codon for VP4 (Met 228) altered to ATA (Ile). Infectious genome production and SV40 particle isolation from African green monkey kidney cells (BS-C-1) (ATCC, http://www.atcc.org/) have been previously reported [8].

### Antibodies, immunoblotting, and trypan blue analysis.

The VP1 and VP2/3 polyclonal antibodies were generous gifts from A. Oppenheim (Jerusalem, Israel). Antibodies against LT were purchased from Calbiochem (http://www.emdbiosciences.com/html/CBC/home.html), Alexa-488-conjugated goat anti-mouse from Molecular Probes (http://probes.invitrogen.com/), and HRP-conjugated goat anti-mouse and anti-rabbit from Amersham (http://www.gelifesciences.com/). The cells were lysed in the dish on ice with 1% NP-40 HBS, 2.4 mM NEM, 50 μM LLnL, 0.4 μM PMSF, and 20 μM leupeptin and collected by scraping. The NP-40 insoluble nuclear fraction was sedimented at 15,000*g* for 5 min and the post-nuclear supernatant was collected. The nuclear fraction was solubilized in 2% SDS, 50 mM Tris (pH 7.5), 1 mM EDTA, 150 mM NaCl, 50 μM LLnL, 0.4 μM PMSF, and 20 μM leupeptin. The lysates were resolved by SDS-PAGE, transferred to PVDF, and subjected to standard immunoblotting. Trypan blue analysis of SV40-infected cells was previously reported [7].

### Cell culture, immunocytochemistry, and microscopy.

BS-C-1 cells were maintained in DMEM supplemented with penicillin-streptomycin and 5% FBS (Invitrogen, http://www.invitrogen.com/) in a humidified 5% CO_2_ incubator at 37 °C. Glass coverslips containing cells were fixed, stained with LT antisera, and analyzed by immunofluorescence microscopy as previously described [7].

### DNA constructs, recombinant protein expression, and purification.

VP1, VP2, VP3, and VP4 were PCR cloned into the pSP72 plasmid (Promega, http://www.promega.com/) for in vitro translations and the pET21d vector (Novagen, http://www.emdbiosciences.com/html/NVG/home.html) for bacterial expression using standard techniques. Site-directed mutagenesis of Met228 within the VP2 reading frame to an Ile was used to create the VP3-M228I and VP3-M228I-His constructs. The expression and purification of VP1Δarm-His using Ni-NTA sepharose and GST-VP3 using GSH-sepharose from the BL21 E. coli Rosetta strain have been previously described [8].

### mRNA synthesis, translations, and pull-down assays.

All pSP72 plasmids containing VP1, VP2, VP3, and VP4 were linearized with the restriction enzyme Nde I, and the pET21d plasmids were linearized with AlwN I. The cDNAs were transcribed with the T7 expression system from Ambion (http://www.ambion.com/) and translated in 10-μl reactions as described previously [25] with 6 mM β-mercaptoethanol used instead of DTT for experiments involving bacterially produced VP1Δarm. For co-translational association with VP1Δarm, the protein was present during synthesis, while post-translational associations involved the addition of the protein after synthesis inhibition. To monitor oligomerization with GST-VP3, the translation reactions were added post-translation to freshly purified GST-VP3 and GST bound to GSH-sepharose in 0.5 ml of PBS (pH 7.3), 0.5% TX-100, and 5 mM DTT. The methods for isolating the complexes of VP1Δarm-His with VP2, VP3, and VP4 by Ni-NTA sepharose have been previously described along with those for isolating bacterial purified GST–VP3 complexes by GSH-sepharose [8].

## Supporting Information

Figure S1 Alignments of VP2 Sequences from JCV and BKV with SV40 by ClustalW The conserved methionines, Met 1 (VP2), Met 119 (VP3), Met 228 (VP4), and Met 295, are indicated. Blank spaces represent no homology, while “*”, “:” and “.” represent identical homology, conserved substitutions, and semi-conserved substitutions, respectively.
(5.0 MB TIF)

Figure S2 VP4 Localizes Primarily to the Nuclear Fraction and Is Degraded by Exogenous Trypsin Immunoblot analysis of post-nuclear and nuclear fractions from BS-C-1 cells transfected with the indicated genomes and harvested in the absence or presence of trypsin. The trypsin-cleaved products of nuclear-localized VP2 and VP3 are noted, and the arrowhead indicates a band with a faster mobility than VP4 that reacts with VP2 and VP3 antisera.
(2.6 MB TIF)

Figure S3 VP4 Has Little Effect on VP3 Association with VP1 Pentamers (A) Purified VP1 pentamers (VP1Δarm-His) were incubated with radiolabled VP3 and VP3-M228I synthesized in reticulocyte lysate co- (Ct) and post (Pt)-translationally prior to isolation. The percentage of the bound (B) fraction from the total (T) is included for two independent experiments.
(B) Controls for GST-VP3 oligomerization. The radiolabeled positive control VP1 and negative control luciferase were synthesized in reticulocyte lystate prior to GST and GST-VP3 binding and isolation. The GST-VP3–bound percentage of the total (T) is included from two independent experiments.
(3.7 MB TIF)

Figure S4 Schematic of the Experimental Protocol Used in Figure 4B E. coli were induced with IPTG (1mM) for 10 min prior to the addition of rifampicin (150 μg/ml) to inhibit transcription of endogenous genes by the E. coli RNA polymerase. The sample was incubated for an additional 30 or 60 min and split (A, B), with one fraction receiving the membrane-impermeable protein synthesis inhibitor hygromycin B (50 μM). The cells were pulsed for 10 min, lysed, and monitored for expression of the individual constructs by SDS-PAGE. The conclusions for the various outcomes are listed.
(5.7 MB TIF)

Figure S5 VP4 Does Not Integrate into the ER Membrane as Efficiently as VP2 and VP3 Radiolabeled constructs were synthesized as in reticulocyte lysate in the presence of VP1Δarm-His. Rough ER microsomes were added post-translationally and a portion of the total lysate (T) was retained. The post-translational integration of the proteins into the ER was determined by co-sedimentation (P) with the rough ER microsomal membranes. The percent of the total for each construct that co-sedimented with the membranes is listed below for two independent experiments.
(2.9 MB TIF)

Figure S6 VP4 Possess a Single Hydrophobic Transmembrane Region Hydrophobicity plot of VP4 using Membrane Protein Explorer 3.0. The predicted transmembrane segment is designated by the line.
(3.2 MB TIF)

### Accession Numbers

The GenBank (http://www.ncbi.nlm.nih.gov/Genbank/) protein sequences from the various polyomaviruses used in this paper are the following:

VP2: African green monkey (NP_848005); baboon (YP_406552); BKV (BAF03118); bovine (NP_040785); budgerigar (NP_848011); crow (YP_529825); finch (ABB04270); goose hemorrhagic (NP_849167); hamster (NP_056734); JCV (AAM89343); murine (NP_041268); murine pneumotropic (NP_041235); and SV40 (AAC59345).

The VP4 nucleotide and protein sequence data reported are available in the Third Party Annotation Section of the DDBJ/EMBL/GenBank databases under the accession number BK006135.

## Abbreviations

ER: endoplasmic reticulum
IPTG: isopropyl-β-D-thiogalactopyranoside
LT: large T antigen
OD: optical density
SV40: simian virus 40
WT: wild-type
